# Later emergence of acquired drug resistance and its effect on treatment outcome in patients treated with Standard Short-Course Chemotherapy for tuberculosis

**DOI:** 10.1186/s12890-016-0187-3

**Published:** 2016-02-04

**Authors:** Jingtao Gao, Yan Ma, Jian Du, Guofeng Zhu, Shouyong Tan, Yanyong Fu, Liping Ma, Lianying Zhang, Feiying Liu, Daiyu Hu, Yanling Zhang, Xiangqun Li, Liang Li, Qi Li

**Affiliations:** Clinical Center on Tuberculosis, Beijing Chest Hospital, Capital Medical University, Beijing Tuberculosis and Thoracic Tumor Research Institute, Beijing, China; State Key Laboratory for Molecular Virology and Genetic Engineering, Institute of Pathogen Biology, Chinese Academy of Medical Sciences & Peking Union Medical College, Beijing, China; Department of TB Control, Guangzhou Chest Hospital, Guangzhou, Guangdong China; Department of TB Control, Tianjin Centers for Disease Control and Prevention, Tianjin, China; Department of TB Control, Henan Center for Disease Control and Prevention, Zhengzhou, Henan China; Department of TB Control, Hebei Center for Disease Control and Prevention, Shijiazhuang, Hebei China; Department of TB Control, Guangxi Center for Disease Control and Prevention, Nanning, Guangxi China; Department of TB Control, Chongqing Anti-tuberculosis Institute, Chongqing, China; Department of TB Control, Yunnan Center for Disease Control and Prevention, Kunming, Yunnan China; Department of TB Control, Shanghai Municipal Center for Disease Control and Prevention, Shanghai, China

**Keywords:** Tuberculosis, Multi-Drug Resistant Tuberculosis, Standard Short-Course Chemotherapy, Acquired drug resistant tuberculosis emergence

## Abstract

**Backgrounds:**

The failure of current Standard Short-Course Chemotherapy (SCC) in new and previously treated cases with tuberculosis (TB) was mainly due to drug resistance development. But little is known on the characteristics of acquired drug resistant TB during SCC and its correlation with SCC failure. The objective of the study is to explore the traits of acquired drug resistant TB emergence and evaluate their impacts on treatment outcomes.

**Methods:**

A prospective observational study was performed on newly admitted smear positive pulmonary TB (PTB) cases without drug resistance pretreatment treated with SCC under China’s National TB Control Program (NTP) condition from 2008 to 2010. Enrolled cases were followed up through sputum smear, culture and drug susceptibility testing (DST) at the end of 1, 2, and 5 months after treatment initiation. The effect factors of early or late emergence of acquired drug resistant TB , such as acquired drug resistance patterns, the number of acquired resistant drugs and previous treatment history were investigated by multivariate logistic regression; and the impact of acquired drug resistant TB emergence on treatment failure were further evaluated.

**Results:**

Among 1671 enrolled new and previously treated cases with SCC, 62 (3.7 %) acquired different patterns of drug resistant TB at early period within 2 months or later around 3–5 months of treatment. Previously treated cases were more likely to develop acquired multi-drug resistant TB (MDR-TB) (OR, 3.8; 95 %CI, 1.4–10.4; *P* = 0.015). Additionally, acquired MDR-TB cases were more likely to emerge at later period around 3-5 months after treatment starting than that of non-MDR-TB mainly appeared within 2 months (OR, 8.3; 95 %CI, 1.7–39.9; *P* = 0.008). Treatment failure was associated with late acquired drug resistant TB emergence (OR, 25.7; 95 %CI, 4.3–153.4; *P* < 0.001) with the reference of early acquired drug resistant TB emergence.

**Conclusions:**

This study demonstrates that later development of acquired drug resistant TB during SCC is liable to suffer treatment failure and acquired MDR-TB pattern may be one of the possible causes.

**Electronic supplementary material:**

The online version of this article (doi:10.1186/s12890-016-0187-3) contains supplementary material, which is available to authorized users.

## Background

TB is a major global health problem with 8.6 million new cases and almost 1.3 million deaths attributed to the disease every year [1]. WHO recommended standardized first-line anti-TB drug regimen is a single 6-month or 8-month regimen composed of isoniazid (H), rifampicin (R), ethambutol (E), and streptomycin (S) with pyrazinamide (Z) [2]. This regimen is recommended for all new TB cases and previously treated cases in more than 90 countries. This strategy is designed and evaluated as the cost-effectiveness regimen used in resource-limited settings for decades for the consideration of convenient treatment management based on same number, dose, and types of medication. Though high cure rate was achieved of SCC for cases with drug-sensitive TB [3], the emergence of drug resistant TB, especially MDR-TB, and acquisition of additional drug resistance during treatment, brought much less efficacy of SCC both in trials [4–7] and under program conditions [8]. Few cohort studies, though the sample size was small, worked on the treatment of mono- or poly-resistant TB with SCC and presented poor results [9]. In most low- and middle-income countries, DST is not routinely performed for new cases nor for most previously treated cases [10, 11]; therefore, cases carrying drug-resistant strains of *Mycobacterium tuberculosis* (MTB) might be at greater risk for SCC failure and disseminating drug resistant strains [12]. China is one of the 27 countries with high MDR-TB burden, early detection and treatment could help prevent its transmission. Currently, China’s NTP [13] provides new and previously treated cases with SCC under directly observed treatment (DOT) and monitors their response to SCC by sputum smear (SS) rather than sputum culture and DST due to limited resources. Sputum culture and DST are recommended to be performed for cases with initial treatment and retreatment failure, where possible, to develop appropriate chemotherapy regimens. Hence, little is known whether drug resistant TB occurs during treatment and few data available links acquired drug resistant TB to treatment failure with SCC. To address these issues, we conducted a prospective observational study tracing the emergence of acquired drug resistant TB at indicated time points among new and previously treated cases receiving first-line standard regimens to determine the characteristics and its effect on treatment failure.

## Methods

### Study settings and population

A prospective and observational cohort study was conducted in 8 provinces including Tianjin, Hebei, Henan, Shanghai, Chongqing, Yunnan, Guangxi, and Guangdong which are situated in northern, eastern, central-western, and southern China from October 2008 to September 2010. This study was embedded in the routine TB control program of each province. Case detection, smear microscopy, chemotherapy and treatment management were routine procedures under NTP; sputum culture and DST at varied time points were added. The sample size for new and previously treated cases of sputum smear-positive (SS+) TB was set at 1927 and 506 respectively which took into account a prevalence of overall resistance to first-line anti-TB drugs (H, R, E, S) of 18.6 % and 46.5 % respectively from a previous study [14], with desired precision of 1 %, a 95 % confidence level and a non-response rate of 10 %, assuming that 15 % of the culture samples would be lost due to failure to recover or due to growth of non-tuberculosis mycobacterium (NTM). Based on the requirements for sample size and the capacity to perform sputum culture and DST, the provincial TB Control and Prevention Centers in the above provinces were selected. The number of cases allocated to each province was based on the number of new SS+ cases reported by that province with the proportion to the total number of cases nationwide in 2007. Study subjects meeting all the following criteria were enrolled: 1. Aged ≥14 years; 2. Informed consent; 3. Newly registered confirmed PTB cases including cases with two positive direct smear microscopy results; or one positive direct smear microscopy result and lung imaging consistent with active PTB imaging manifestations [13]. 4. Not NTM strains infection; 5. Strains susceptible to all 4 first line anti-TB drugs (H, R, E, S) pretreatment (month 0). Demographic information and relevant medical history including age, gender, physical examinations, previous anti-TB treatment history and contact history with TB index cases were collected with a standard questionnaire given upon study entry. SS, culture, and DST results were examined at month 1, 2, and 5. Treatment outcomes were evaluated for both pan-susceptible cases all through SCC and acquired drug resistant TB cases during SCC.

### Definitions

New cases of TB were defined as those who had never been treated for TB or had taken anti-TB drugs for less than one month. Previously treated cases were defined as those who had been previously treated for one month or more with anti-TB drugs [13]. In our study, according to the time of acquired drug resistant TB emergence for the first time, cases with any drug resistant TB emergence before the end of 2 months after treatment initiation were defined as early emergence of drug resistance while cases with drug resistant TB emergence after 2 months were defined as late emergence of drug resistance. Treatment outcomes were defined according to the WHO guidelines [2]. A patient who was initially SS+ and who was sputum smear-negative in the last month of treatment and on at least one previous occasion was defined as “Cured”. A patient who completed treatment but did not meet the criteria for cure or failure was defined as “Completed treatment”. A patient who died from any cause during treatment was defined as “Died”. A patient who was initially SS+ and who remained SS+ at month 5 or later during treatment was defined as “Failed”. A patient whose treatment was interrupted for two consecutive months or more was defined as “Defaulted”. A patient whose treatment outcome was not known was defined as “Not evaluated”. For analysis, a patient who was cured or who completed treatment was combined as “Successfully treated”. In this study we mainly focus on cases with treatment success and failure of SCC.

### Treatment regimen

All cases with pan-susceptible MTB strains prior treatment received SCC under qualified DOT. The initial standard regimen was 2H_3_R_3_Z_3_E_3_/4H_3_R_3_, and the retreatment standard regimen was 2H_3_R_3_Z_3_E_3_S_3_/6H_3_R_3_E_3_ [13]. For patients who could not use streptomycin for any reason, the intensive phase was extended for an additional month as 3H_3_R_3_Z_3_E_3_/6H_3_R_3_E_3._ The following regimen modifications were made after the second month SS examination: First, if a new SS+ TB patient was still tested SS+ at the end of the second month, the intensive phase was extended for an additional month, and the continuation phase remained unchanged. A smear microscopy was performed at the end of the third month. If the SS was negative at the end of the fifth month, the treatment regimen was 3H_3_R_3_Z_3_E_3_/4H_3_R_3_. Second, if a retreatment SS + TB patient had a positive SS at the end of the second month, the intensive phase was extended for an additional month if the treatment regimen contained streptomycin and the continuation phase remained unchanged as 3H_3_R_3_Z_3_E_3_S_3_/6H_3_R_3_E_3_, or the intensive phase was extended for another month, if the treatment regimen did not contain streptomycin, and the continuation phase remained unchanged as 4H_3_R_3_Z_3_E_3_/6H_3_R_3_E_3_. In both cases, a smear microscopy was performed at the end of the third month.

### Bacteriologic examination

Three consecutive sputum samples (spot, night, and morning) were collected before the initiation of treatment (month 0) and 2 sputum samples were collected at months 1, 2, and 5 for each eligible patient. For isolation of the culture, each specimen was decontaminated, digested, and homogenized using the standard Petroff method [15]. An aliquot of 0.1 ml of the resulting specimen was inoculated into acidified Löwenstein–Jensen (LJ) slant tubes for primary isolation of the organism. Tubes were then incubated at 37 °C and inspected for growth of *Mycobacterium* for a period of 8 weeks. If no bacteria grew by week 8, the result was recorded as negative. Cultures with growing colonies were continued for identification and DST. Susceptibility testing of TB isolates to four first-line antimicrobial agents was performed by the indirect proportion method with LJ medium. The following drug concentrations were used to distinguish resistant isolates from susceptible isolates: isoniazid (H, 0.2 μg/mL), rifampicin (R, 40 μg/mL), ethambutol (E, 2 μg/mL), and streptomycin (S, 4 μg/mL). A strain was considered resistant when bacterial growth on a drug-containing medium was equal to or greater than 1 % of the colonies that grew on a drug-free medium. Quality-assured DST is critical to ensure accurate detection of drug resistance for subsequent treatment decisions and to avoid false diagnosis. All laboratories engaging in this study had external quality assessment coverage of DST. Internal control of sensitivity testing was assessed using a MTB sensitive strain (H37Rv).

### Ethical considerations

This study was reviewed and approved by the Institutional Ethics Review Committee of Beijing Chest Hospital, Capital Medical University, Beijing Tuberculosis and Thoracic Tumor Research Institute, and selected 8 provincial TB Control and Prevention Centers. Written informed consent was obtained from all SS+ cases before enrollment in the study. In particular, for the minors enrolled in the study, written informed consent was obtained from themselves and their parents or guardians before enrollment. DST results were promptly reported back to the respective local health facilities the patients visited for further treatment management from above selected areas.

### Statistics

The categorical variables were analyzed using Pearson’s Chi-square test or Fisher’s exact test as appropriate. To evaluate the effect factors of acquired drug resistant TB emergence, variables significant (*p* < 0.05) in the univariate analysis were subsequently analyzed by multiple logistic regression with a stepwise and forward method to identify the statistically significant factors to be maintained in the final model. For exploring the effect of acquired drug resistant TB emergence on treatment failure, we compared cases with late emergence to those with early emergence using univariate analysis by computing odds ratios (ORs) and their 95 % confidence intervals (CI). Data analysis was performed using SPSS version 18.0 (SPSS, Chicago). *P* value <0.05 was considered as criterion for statistical significance.

## Results

### Patient enrollment and demographic characteristics

**Fig. 1:**
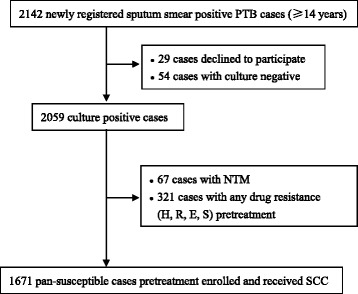
Flow chart of TB subjects enrolled in the study

During the study period, among 2142 confirmed pulmonary TB cases detected, 67 cases infected with NTM, 29 cases declined to participate the study, 321 cases with drug resistant TB and 54 cases with negative sputum culture results before treatment initiation. Therefore, according to the inclusion and exclusion criteria, the above cases were excluded and a total of 1671 pan-susceptible TB cases to all four first-line anti-TB drugs confirmed by pretreatment DST were enrolled and then received standardized anti-TB treatment (Fig. 1). Of these 1671 pan-susceptible cases pretreatment, 62 cases developed drug resistant TB during anti-TB therapy. Table 1 showed the analysis of univariate risk factors for acquired MDR-TB and Non-MDR-TB respectively. Non-MDR-TB refers to the cases with drug resistance other than MDR-TB. We found those aged 40–59 years (OR, 17.5; 95 %CI, 2.3–134.6; *P* < 0.001) was a significant factor for acquired MDR-TB development. Cases who had received previous TB treatment (OR, 3.8; 95 %CI, 1.4–10.4; *P* = 0.015) were more likely to develop acquired MDR-TB.

**Table 1:** Demographic characteristics and risk factors for patients with acquired drug resistant TB during SCC

Characteristics	Pan-susceptible TB throughout^a^ n = 1609(%)	Any acquired drug resistant TB^b^		Acquired MDR-TB vs. Pan-susceptible TB throughout		Acquired Non- MDR-TB vs. Pan-susceptible TB throughout	
		MDR-TB^c^ n = 17(%)	Non-MDR-TB^d^ n = 45(%)	OR (95 % CI)^e^	*P* value	OR(95 % CI)	*P* value
Age(years)							
<40	756(47.0)	1(5.9)	19(42.2)	Reference		Reference	
40–59	560(34.8)	13(76.5)	20(44.5)	17.5(2.3–134.6)	<0.001	1.4(0.8–2.7)	0.278
≥60	293(18.2)	3(17.6)	6(13.3)	7.7(0.8–74.7)	0.07^g^	0.8(0.3–2.1)	0.665
Gender							
Male	1160(72.1)	14(82.4)	35(77.8)	Reference		Reference	
Female	449(27.9)	3(17.6)	10(22.2)	0.6(0.2–1.9)	0.427^g^	0.7 (0.4–1.5)	0.401
BMI^f^ (Kg/m^2^)							
BMI < 18.5	620(38.5)	3(17.6)	18(40.0)	Reference		Reference	
BMI ≥ 18.5	989(61.5)	14(82.4)	27(60.0)	2.9(0.8–10.2)	0.078	0.9(0.5–1.7)	0.842
Treatment history							
New	1407(87.4)	11(64.7)	38(84.4)	Reference		Reference	
Previously treated	202(12.6)	6(35.3)	7(15.6)	3.8(1.4–10.4)	0.015^g^	1.3(0.6–2.9)	0.550

^a^Pan-susceptible TB throughout in our study was defined as cases with TB strains susceptible to all four first-line anti-TB drugs including soniazid(H), rifampicin(R), ethambutol(E), and streptomycin(S) both pretreatment and throughout therapy

^b^Any acquired drug resistant TB in our study was defined as cases with TB strains that were susceptible to all four first-line anti-TB drugs pretreatment, but developed resistance to at least one of the four drugs during SCC

^c^ MDR-TB was defined as TB that was at least resistant to both isoniazid and rifampicin

^d^Non-MDR-TB in our study was defined as TB cases with any drug resistance pattern except MDR-TB

^e^OR, odds ratio. CI, confidence interval

^f^BMI, body mass index, calculated as the weight in kilograms divided by the square of the height in meters

^g^ *P* value was determined by Fisher’s Exact Test. Not indicated was determined by Pearson’s Chi-Square Test

### Acquired drug resistant TB emergence and its characteristics

Before starting treatment, 321 cases were resistant to at least one of the four first-line anti-TB drugs (H, R, E, and S), of which 251 were new and 70 were previously treated; 1671 cases were susceptible to all four drugs tested, of which 62 acquired drug resistant TB during SCC, composed of 49 new and 13 previously treated cases. The overall prevalence of drug resistance in our study was 17.6 % (300 of 1707) of new cases and 29.1 % (83 of 285) of previously treated cases. Furthermore, the prevalence of MDR-TB was 2.6 % (45 of 1707) of new cases and 14.0 % (40 of 285) of previously treated cases in the study (Additional file 1: Table S1). For the 62 acquired drug resistant TB cases, 89.8 % of new (44 of 49) and 61.5 % (8 of 13) of previously treated cases developed drug resistance within 2 months of SCC. In addition, 75.0 % newly acquired drug resistant TB (39 of 52) cases were resistant to 1 or 2 anti-TB drugs within first 2 months, while 70.0 % newly acquired drug resistant TB cases were resistant to 3 or 4 anti-TB drugs during 3–5 months after treatment starting. The acquired drug resistance patterns appeared within 2 months were mainly non-MDR-TB, accounted for 80.8 % (42 of 52) among patients with acquired drug resistance of same period, while 3–5 months after treatment starting, 70.0 % (7 of 10) newly acquired drug resistant TB cases were of MDR-TB pattern. In multivariate logistic regression analysis, late emergence of acquired drug resistant TB was associated with drug resistance patterns. Acquired MDR-TB cases were more likely to emerge at later period than that of acquired non-MDR-TB cases (OR, 8.3; 95 %CI, 1.7–39.9; *P* = 0.008) (Table 2).

**Table 2:** Frequency and effect factors of acquired drug resistant TB emergence at varied time points during SCC

Variable	Month1(1) n = 35 (%)	Month2(2) n = 17 (%)	Month3-5(3) n = 10 (%)	Univariate analysis (3) vs (1 + 2)	Multivariate logistic regression (3) vs (1 + 2)	
				*P* value	OR(95 % CI)	*P* value
Treatment history				0.027^*^		
New	32 (91.4)	12 (70.6)	5 (50.0)			
Previously treated	3 (8.6)	5 (29.4)	5 (50.0)			
No. of drugs to which isolate is resistant				0.015^*^		
1	21(60)	6(35.3)	2(20.0)			
2	9(25.7)	3(17.6)	1(10.0)			
3	4(11.4)	5(29.5)	5(50.0)			
4	1(2.9)	3(17.6)	2(20.0)			
Drug resistance patterns				0.003^*^		
Non-MDR-TB	30 (85.7)	12 (70.6)	3 (30.0)		Reference	
MDR-TB	5 (14.3)	5 (29.4)	7 (70.0)		8.3(1.7–39.9)	0.008

^*^ *P* value was determined by Fisher’s Exact Test

### Treatment outcomes of acquired drug resistant TB cases with standardized regimen

84.3 % of cases with pan-susceptible TB throughout were successfully treated and similar treatment success rate was achieved of cases with acquired drug resistant TB within 2 months of SCC, while only 20 % cases with late drug resistant TB emergence after 2 months were successfully treated (Table 3). The impact of time points of acquired drug resistant TB emergence on treatment outcomes evaluated indicated that treatment failure was significantly more likely among late emergence cases than among early emergence cases (OR, 25.7; 95 %CI, 4.3–153.4; *P* < 0.001) (Table 4). In addition, we analyzed the treatment outcomes of 62 acquired drug resistant cases during SCC based on drug resistance patterns. The treatment success rate of acquired MDR-TB cases was 52.9 %, much lower than that with non-MDR-TB pattern at 82.2 %.We further analyzed treatment success rate of 3 subgroups of non-MDR-TB. It is reported 92.3 % cases with any H resistance and 89.5 % cases with E/S resistance were successfully treated respectively. However, the treatment success rate of cases with any R resistance was 61.5 % (Additional file 1: Table S2).

**Table 3:** Treatment outcomes of both pan-susceptible TB cases throughout SCC and those acquired drug resistant TB during treatment

Treatment outcomes n(%)	Pan-susceptible TB throughout^a^ N = 1609	Acquired drug resistant TB N = 62		
		≦2 months	3–5 months	Total
Successfully treated	1357(84.3)	44(84.6)	2(20.0)	46(74.1)
Failed	102(6.3)	6(11.5)	7(70.0)	13(21.0)
Died	36(2.2)	0(0.0)	1(10.0)	1(1.6)
Defaulted	39(2.4)	1(1.9)	0(0.0)	1(1.6)
Not evaluated	75(4.7)	1(1.9)	0(0.0)	1(1.6)

^a^Pan-susceptible TB throughout in our study was defined as cases with TB strains susceptible to all four first-line anti-TB drugs including isoniazid(H), rifampicin (R), ethambutol (E), and streptomycin (S) both pretreatment and throughout the therapy

**Table 4:** Effect of acquired drug resistant TB emergence during SCC on treatment failure

Time of acquired drug resistance emergence	Total	Treatment success n(%)	Treatment failure n(%)	Univariate analysis	
				OR(95 % CI)	*P* value
≦2 months	52	44(84.6)	6(11.5)	Reference	<0.001^*^
3–5 months	10	2(20.0)	7(70.0)	25.7(4.3–153.4)	

^*^ *P* value was determined by Fisher’s Exact Test

## Discussion

Up to date, this study is the first to evaluate traits of acquired drug resistant TB emergence and its impact on treatment failure of SCC under China’s routine NTP condition. Majority of acquired drug resistant TB cases developed early within 2 months with SCC and late acquired drug resistant TB emergence after 2 months is more likely associated with the drug resistance pattern of MDR-TB. Furthermore, our results indicated a good response to SCC in cases with pan-susceptible strains throughout therapy which reconfirmed the SCC effectiveness for cases with susceptible strains [8, 16]. However, our findings presented poor response of SCC in cases with acquired drug resistant TB, especially with late drug resistant TB. It is shown that late acquired drug resistant TB emergence after 2 months is 25.7 times higher to result in treatment failure than that within 2 months.

In our study, the data from 8 provinces in China indicating a overall drug resistant TB prevalence of 17.6 % in new cases and 29.1 % in previously treated cases and a MDR-TB prevalence of 2.6 % and 14.0 % in new and previously treated cases respectively are lower than that from China’s national drug resistance survey in 2007 [17]. The reduction of drug resistant TB and MDR-TB prevalence in our study could be explained by two possible reasons. Firstly, given the prevalence of TB and MDR-TB not balanced which was highest in western China, 2 provinces from 4 main areas in the study may not be representative of the overall situation in China. Secondly, the drug resistant TB cases in the study were from TB Control and Prevention Centers system not TB specialized hospitals system where more previously treated cases tend to access for further second line anti-TB drugs due to MDR-TB pretreatment [18].

It is well known that previous TB treatment is a strong determinant of drug resistance [17, 19–22]. Current data suggests that previously treated cases are more likely to develop acquired drug resistance, especially MDR-TB, during therapy than that of new cases. However, it is impossible for us to determine the identified reason of acquired drug resistant TB during SCC without DNA fingerprinting analysis of resistant strains compared to that of original strains pretreatment. This represents a limitation of the study. Two reasons might contribute to the acquired drug resistant TB emergence under qualified DOT and quality-assured drug apply under China’s NTP. First, the selection of resistant mutants in mixed bacterial population infected pretreatment due to killing of susceptible strains by anti-TB drugs of SCC. Second, infection of new drug resistant strains may be another explanation for the acquired drug resistant TB emergence during SCC [23].

Several researchers have reported that amplification of resistance to additional anti-TB drugs while receiving WHO recommended SCC [24]. Few data was reported on the traits of acquired drug resistant TB emergence receiving SCC with susceptible strains pretreatment. However, it is important to identify drug-resistant cases in time with standard treatment and prevent its dissemination. We found the time point of acquired drug resistant TB emergence was associated with drug resistance patterns. Cases with MDR-TB development were 8.3 times more likely to be late emergence compared to the non-MDR-TB pattern. Moreover, the cases with late emergence of acquired drug resistance are of high risk to contribute to SCC failure. In line with other studies, anti-TB drug resistance especially MDR-TB has a negative impact on treatment outcome of SCC [8, 12, 16, 25–29]. Treatment success rate of MDR-TB cases was 58 % in Peru and 60 % in Hongkong with SCC [8]. One study in rural counties of eastern China indicated that the cure rate of MDR-TB and other drug resistant TB were 58.3 % and 91.0 % of SCC [30]. Different from these studies, we excluded the drug resistant TB cases pretreatment to target on the characteristics of drug resistant TB emergence during SCC and further explore its impact on treatment outcome. Treatment success rate of cases with acquired MDR-TB was 52.9 % while 82.2 % with non-MDR-TB, a little lower than that in Peru, Hongkong and eastern China. We also analyzed treatment success rate of 3 subgroups of non-MDR-TB indicating that cases with any H resistance and E/S resistance have higher treatment success rate around 90 % while much lower treatment success rate of 61.5 % with any R resistance with SCC. Some discordant impacts of drug susceptibility patterns on treatment success are reported in the literature [25, 31, 32]. This could be explained by the different target population in our study. The greatest finding of this study is that the time point of acquired drug resistant TB emergence significantly impacted treatment outcomes with SCC. Cases with acquired drug resistant TB at 3–5 months were 25.7 times higher (OR, 25.7; 95 %CI, 4.3–153.4; *P* < 0.001) than that of cases with acquired drug resistant TB within 2 months of SCC to experience treatment failure.

Besides drug resistant TB emergence time point, many other factors including low BMI, smoking, and some behaviors such as alcohol consumption and drug abuse are also associated with poor treatment outcomes [33–36]. Diabetes and baseline disease severity of TB also have been shown to be independent risk factors for poor treatment outcomes in previous studies [18, 26, 37–39].

As reported, the use of standard first-line anti- TB treatment on cases with drug resistant TB pretreatment have greater likelihood to get relapse, treatment failure and acquired drug resistance [12, 40–42]. Therefore, pretreatment DST were carried out for individual patients and those with any drug resistance were excluded from our study and transferred to a DOTS-Plus program with the consideration of providing a more tailored regimen for optimal treatment outcomes. Without doubt, DST performed before and during SCC could provide information to recognize drug resistant TB, particularly MDR-TB. Our studies suggested DST should be taken before treatment starting and subsequent DST should be checked regarding patients who remain bacteriological positive at the month 2 or 3 and it is better to provide accordingly effective regimen rather than keeping using SCC in the setting with drug resistant TB emergence later after 2 months as these cases are more likely to fail with SCC. The pressing need to prevent MDR-TB warrants this recommendation and this approach will help decrease its transmission.

Our study has several limitations. First, we did not provide longer follow-up information to evaluate recurrence rates and correlated risk factors for long-term prognosis among successfully treated acquired drug-resistant TB cases. Second, this study was limited by the relatively small number of acquired drug resistant TB cases during SCC. A larger sample could better present the association of treatment failure and its impact factors. Hence, larger scale cohort studies are still needed to further verify the findings of our study. Third, we were not able to measure certain factors possibly related to treatment failure, for example, comorbidities including diabetes, chronic obstructive pulmonary disease, chronic hepatitis, and bacterial load. Fourth, our study is lack of DNA fingerprinting examination to differentiate the origin of acquired drug resistant TB.

Despite these limitations, to date, this might be the first study to evaluate the characteristics of acquired drug resistant TB and its effect on treatment failure with SCC. This study demonstrated later emergence of acquired drug resistant TB during SCC is the prognostic risk factor for treatment failure. Our findings may help relevant policy makers to take more consideration of treatment management on TB cases with potential failed outcomes. Early detection of treatment failure will decrease transmission and decrease likelihood of additional drug resistance acquisition, providing more probability to choose effective regimen. Administration of effective regimen may optimize cure rates and drug resistance acquisition may be avoided. Rapid, feasible and economical culture and molecular biology methods such as GeneXpert are imperative to be applied for identifying drug resistant TB in time pretreatment or during SCC. Effective and comprehensive TB control strategies with adapted DOT is needed to prevent drug resistance especially MDR-TB development. More strict infection control and health education measures should be taken to minimize the transmission of TB and drug resistant TB bacilli in public and ensure patients adherence to treatment preventing drug resistance development. In addition, we expect more robust predictors developed which could evaluate factors that could affect underlying pathological process of the disease being treated and measure the effects of interventions on clinical outcomes in multiple aspects [43].

## Conclusion

Later emergence of acquired drug resistant TB during SCC is prognostic risk factor for treatment failure. Early detection of treatment failure will decrease transmission and decrease likelihood of additional drug resistance acquisition, providing more probability to choose effective regimen.

## Additional file

Additional file 1: Table S1. Drug resistant TB and MDR-TB prevalence among new and previously treated cases of the study. **Table S2.** Treatment success rate of acquired drug resistant TB stratified by drug resistance patterns. (DOCX 41 kb)

## Abbreviations

DOT: directly observed treatment
DST: drug susceptibility testing
E: ethambutol
H: isoniazid
LJ: Löwenstein–Jensen
MDR-TB: multi-drug resistant TB
MTB: mycobacterium tuberculosis
NTM: non-tuberculosis mycobacterium
NTP: national TB control program
OR: odds ratios
PTB: pulmonary TB
R: rifampicin
S: streptomycin
SCC: standard short-course chemotherapy
SS: sputum smear
SS+: sputum smear-positive
TB: tuberculosis
Z: pyrazinamide
