# Supplementary material for: Later emergence of acquired drug resistance and its effect on treatment outcome in patients treated with Standard Short-Course Chemotherapy for tuberculosis
Source: BMC Pulm Med. 2016 Feb 4;16:26. doi: 10.1186/s12890-016-0187-3 (PMC4743330; doi:10.1186/s12890-016-0187-3)
Supplement: Additional file 1: Table S1. — Drug resistant TB and MDR-TB prevalence among new and previously treated cases of the study. Table S2. Treatment success rate of acquired drug resistant TB stratified by drug resistance patterns. (DOCX 41 kb) [file 12890_2016_187_MOESM1_ESM.docx]

**Table S1. Drug resistant TB and MDR-TB prevalence among new and previously treated cases of the study (n=1992).**

| Treatment classification | Pan-susceptible TB throughout | Drug resistant TB ^a^ (and MDR-TB) | | |
| --- | --- | --- | --- | --- |
|  |  | Pretreatment | During SCC | Sub-Total |
| New | 1407 | 251(34) | 49(11) | 300(45) |
| Previously treated | 202 | 70(34) | 13(6) | 83(40) |
| Total | 1609 | 321(68) | 62(17) | 383(85) |

^a^ Drug resistant TB here included drug resistant patients pretreatment and patients with acquired drug resistance during SCC period who were confirmed as pan-susceptible TB pretreatment.

| **Drug resistance patterns** | Total | Treatment success n（%） | Treatment failure n（%） |
| --- | --- | --- | --- |
| MDR-TB | 17 | 9(52.9) | 7(41.2) |
| Non-MDR-TB | 45 | 37(82.2) | 6(13.3) |
| Any R resistance | 13 | 8(61.5) | 4(30.8) |
| Any H resistance | 13 | 12(92.3) | 0(0) |
| E/S resistance ^a^ | 19 | 17(89.5) | 2(10.5) |

**Table S2. Treatment success rate of acquired drug resistant TB stratified by drug resistance patterns (n=62).**

^a^ E/S resistance denoted TB strains resistant to ethambutol and/or streptomycin and susceptible to isoniazid and rifampicin.
